# Acute Pericardial Effusion Revealing Cardiac Sarcoidosis: A Case Report

**DOI:** 10.7759/cureus.106650

**Published:** 2026-04-08

**Authors:** Sidi Mohamed Limame, Sidi Oumar Mohamed Lemine, Mohamed Abdellahi Deddah, Marega Thierno, Chighaly El Hadj Sidi

**Affiliations:** 1 Cardiology, Centre National de Cardiologie, Nouakchott, MRT; 2 Pulmonology, Centre Hospitalier National, Nouakchott, MRT; 3 Pathology, National Oncology Center, Nouakchott, MRT; 4 Cardiovascular and Thoracic Surgery, Centre National de Cardiologie, Nouakchott, MRT

**Keywords:** acute pericarditis, dyspnea, pericardial biopsy, sarcoidosis, surgical pericardial drainage

## Abstract

Sarcoidosis is a rare disease of unknown cause. Several pathophysiological hypotheses have been proposed, including possible infectious or environmental triggers, genetic exposures, and autoimmune responses. Cardiac sarcoidosis preferentially affects the myocardium. We report the case of a 58-year-old patient with a history of hypertension who presented with acute pericarditis with effusion. The patient underwent surgical pericardial drainage. Analysis of the pericardial fluid showed a hemorrhagic and exudative appearance, with a protein level of 52 g/L (reference ˂30 g/L) and hypercellularity, predominantly lymphocytes at 93% (reference ˂50%). Pericardial fluid culture was negative, and PCR did not detect *Mycobacterium tuberculosis* DNA. Pericardial biopsy revealed a giant cell granuloma without caseous necrosis. This report describes a rare presentation of sarcoidosis and highlights the importance of a thorough etiological workup, particularly pericardial biopsy, in the investigation of acute pericarditis.

## Introduction

Sarcoidosis is a rare granulomatous disease of unknown cause. It affects all organs and most often manifests at an average age of 55 years. Its incidence is around 10 per 100,000 person-years in the United States, with a predominance in African Americans [1]. This disease is characterized by the formation of immune granulomas, complex structures composed of monocytes, macrophages, and T lymphocytes. These granulomas result from an exaggerated immune response triggered by an unidentified agent, occurring at a specific time and in conjunction with particular environmental factors in a genetically predisposed individual [2].

Cardiac sarcoidosis is detected on imaging in approximately 20% of patients with systemic sarcoidosis. The clinical presentations of cardiac sarcoidosis are variable [3]. It most commonly mimics moderately hypertrophied cardiomyopathy or arrhythmogenic right ventricular dysplasia [4]. Pericardial involvement is exceptionally rare, representing less than 1% of cardiac presentations, and typically manifests as a minimal pericardial effusion [5]. The diagnosis of sarcoidosis is not standardized but is based on three major criteria: a compatible clinical and/or radiological presentation, histological evidence of non-necrotizing granulomatous inflammation in one or more tissues, and the exclusion of alternative causes of granulomatous disease [6]. Sarcoidosis is associated with high morbidity and mortality in African Americans [7], and a higher prevalence of advanced disease and familial hypercholesterolemia has been reported among Black patients with sarcoidosis [8].

The treatment of sarcoidosis relies on corticosteroid therapy as the first-line approach, sometimes combined with methotrexate. Anti-tumor necrosis factor agents are reserved for refractory cases [9]. In cases of cardiac sarcoidosis, assessment of arrhythmic risk guides the decision for device therapy [1]. In this report, we describe a patient presenting with an acute pericardial effusion that revealed isolated cardiac sarcoidosis. This case illustrates a rare presentation of sarcoidosis and highlights the importance of a thorough etiological workup, particularly pericardial biopsy, in the evaluation of acute pericarditis.

## Case presentation

**Figure 1:**
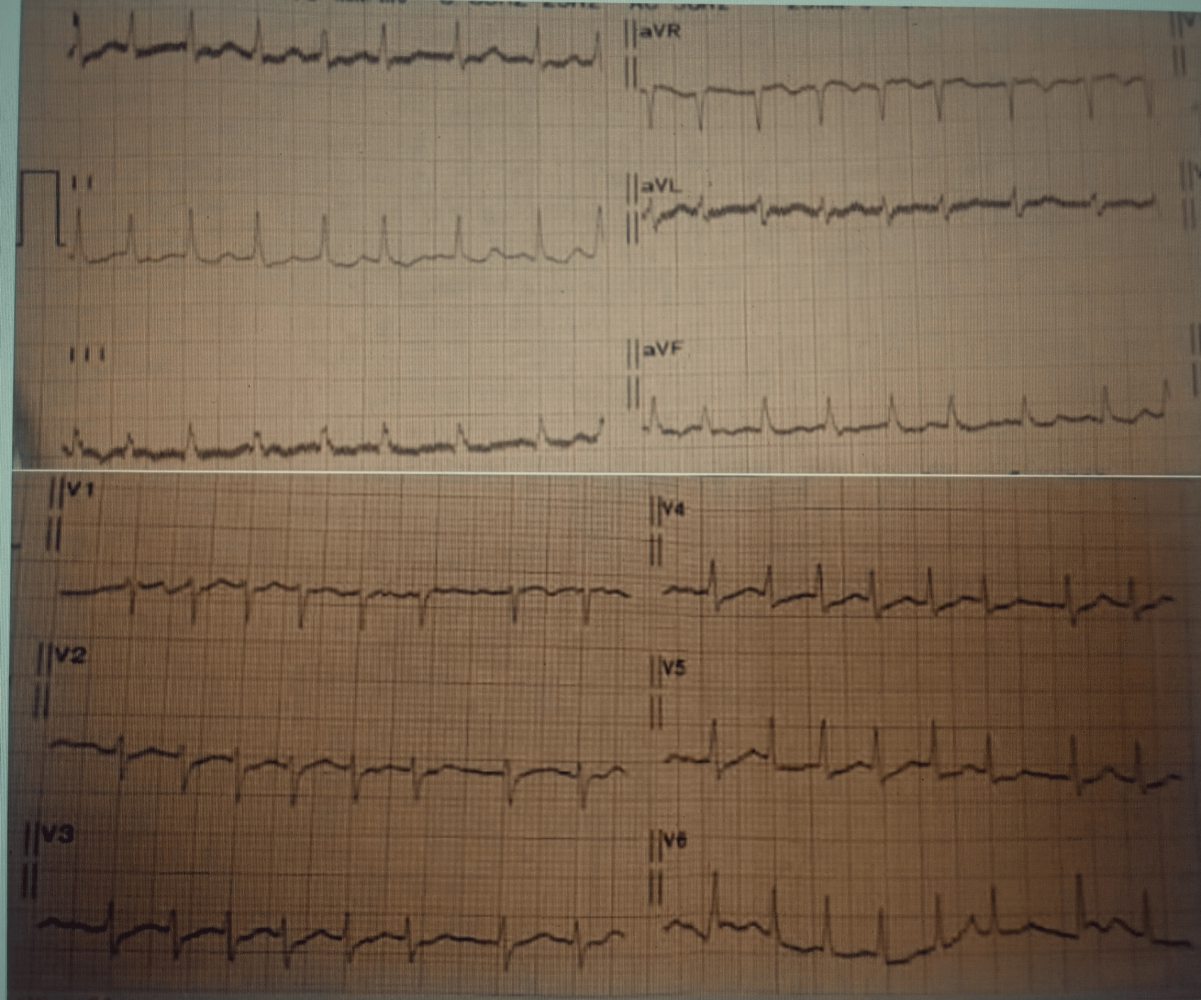
Electrocardiogram The image shows complete tachyarrhythmia due to atrial fibrillation, low voltage, and diffuse negative T waves

**Figure 2:**
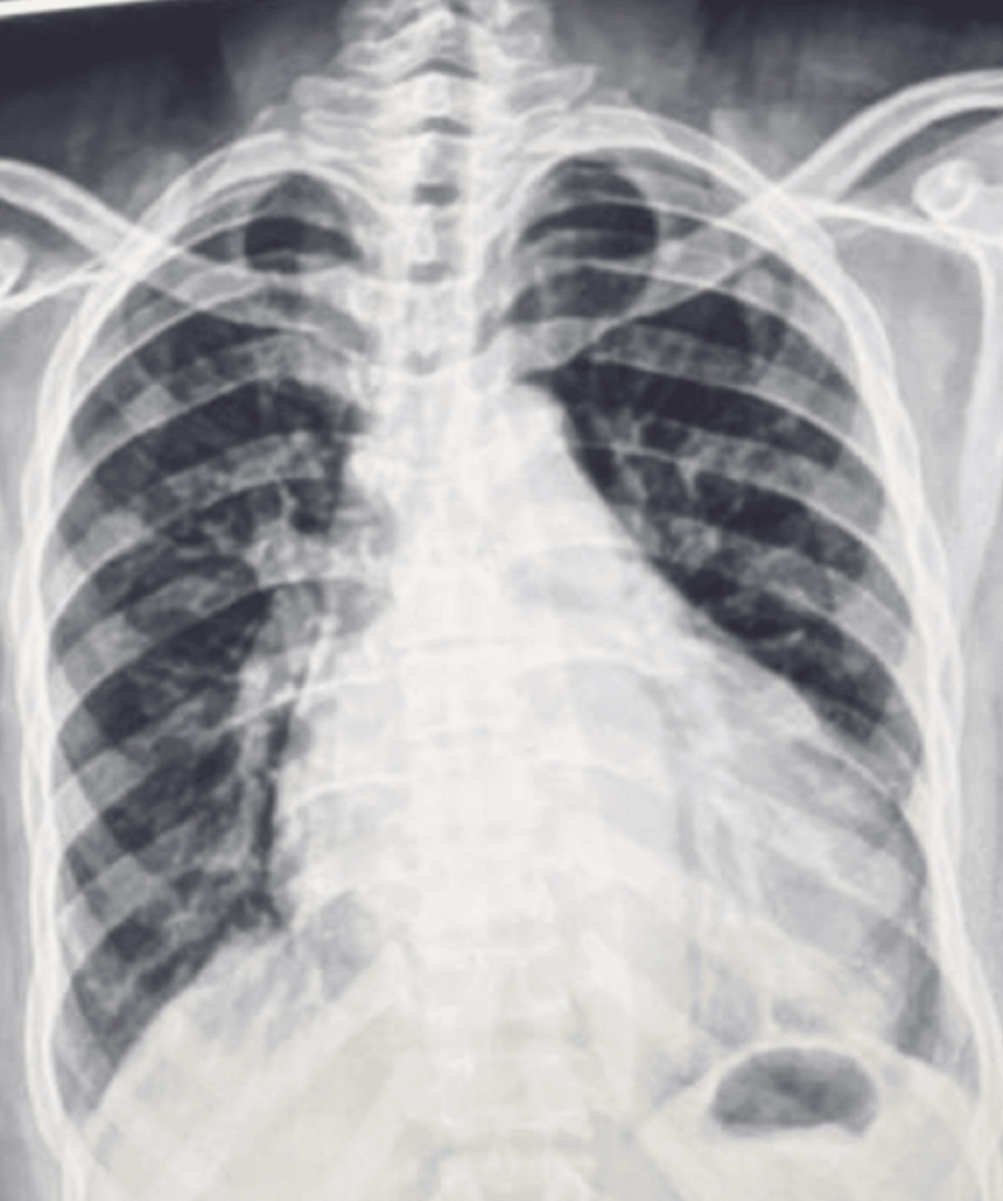
Chest X-ray showing cardiomegaly

**Figure 3:**
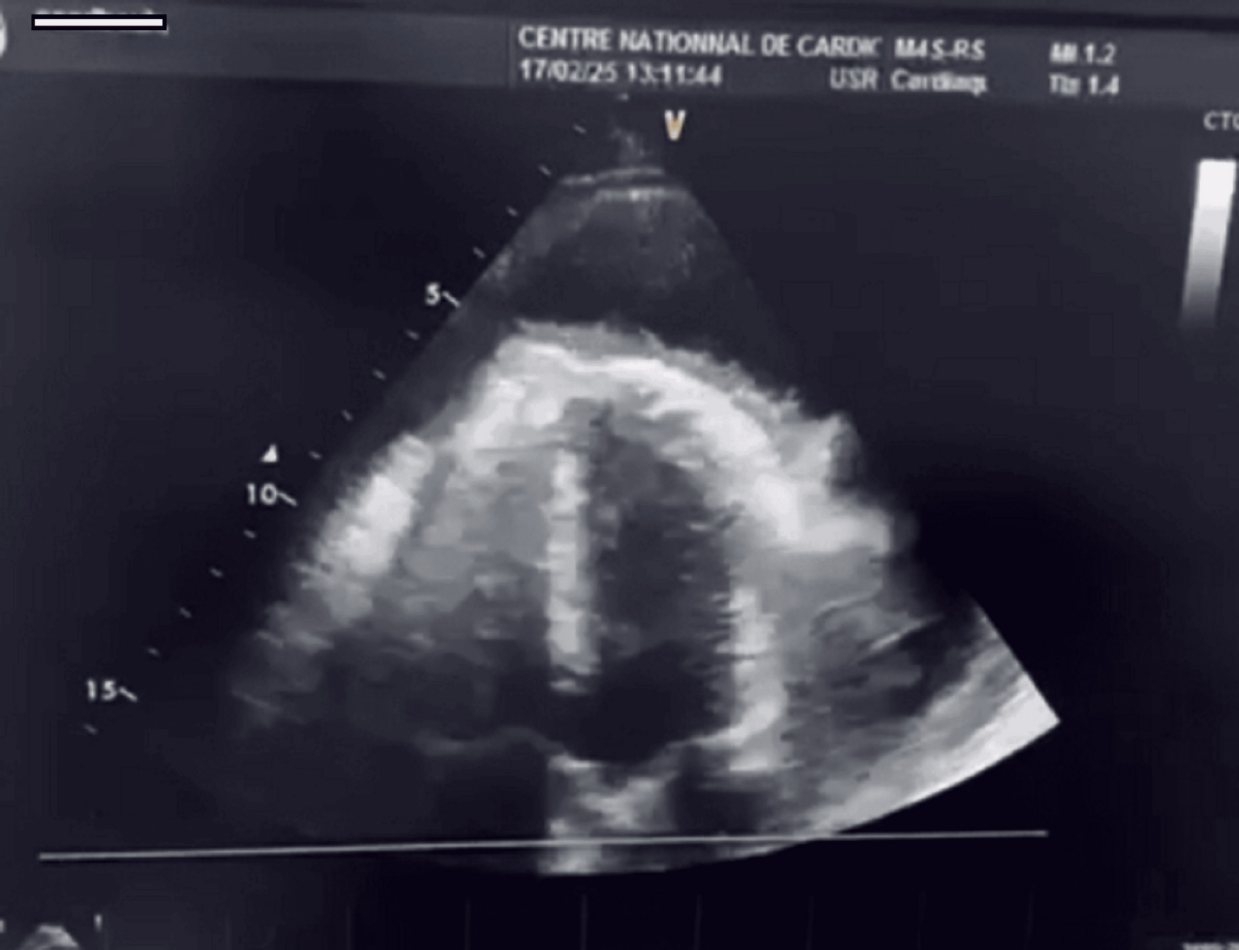
Transthoracic echocardiography The image shows a large circumferential pericardial effusion measuring 24 mm with signs of impending compression of the right heart chambers

The patient was a 58-year-old male with a history of hypertension. He consulted at the National Cardiology Center in Nouakchott, Mauritania, due to the progressive onset of dyspnea at rest over the past week. He had no associated flu-like symptoms or chest pain. Physical examination revealed a blood pressure of 120/80 mmHg, a heart rate of 157 bpm, an oxygen saturation of 99% in room air, and a respiratory rate of 20 breaths/min. He had spontaneous jugular venous distension and muffled heart sounds. His lung fields were clear. The electrocardiogram showed complete tachyarrhythmia due to atrial fibrillation, low voltage, and diffuse negative T waves, changes compatible with a massive effusion dampening the electrical signal (Figure 1). The chest X-ray showed cardiomegaly (Figure 2). Transthoracic echocardiography revealed a large circumferential pericardial effusion measuring 24 mm with signs of impending compression of the right heart chambers. The left ventricle was of normal size and morphology with preserved function. The valves were normal (Figure 3).

**Figure 4:**
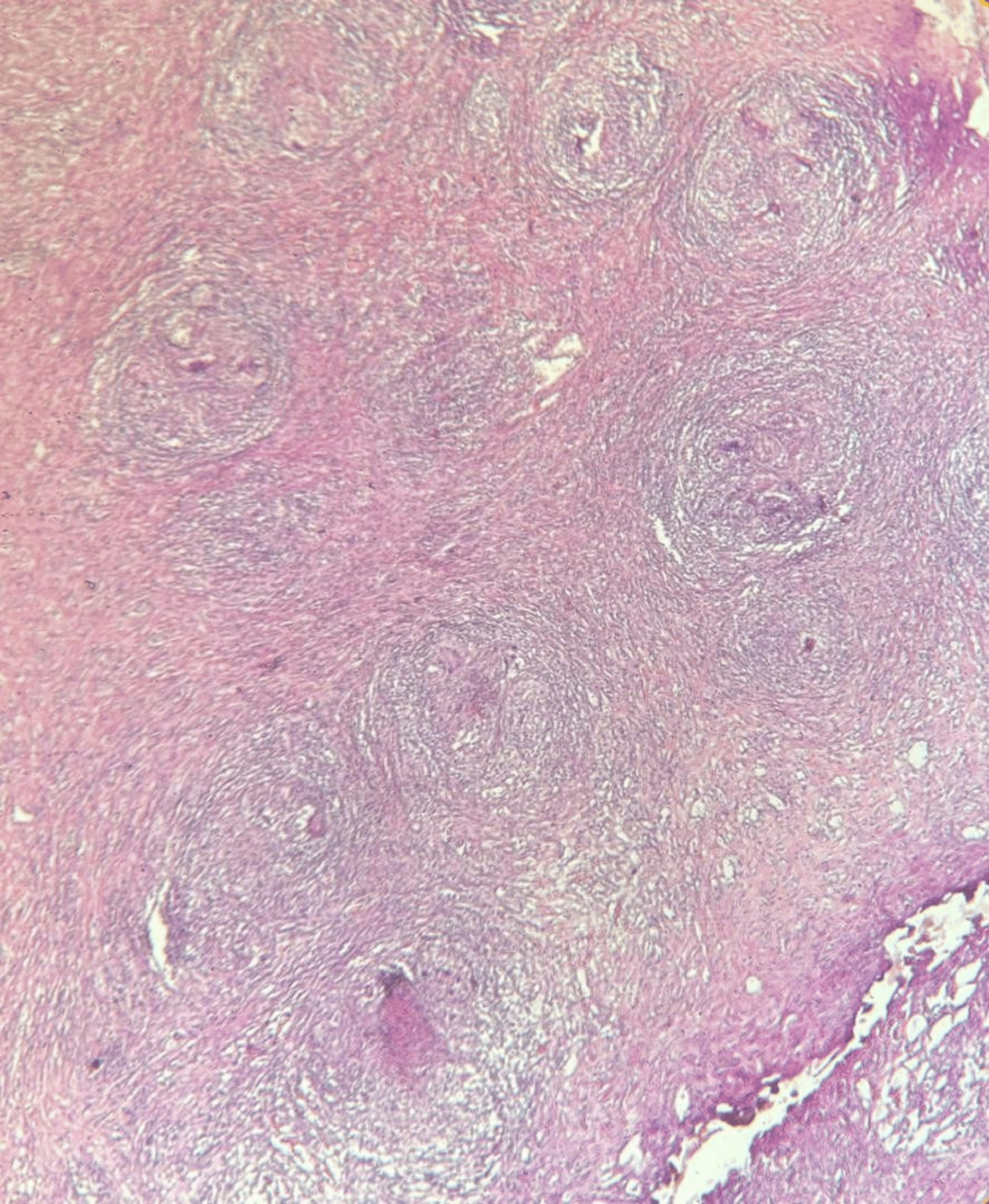
Low-magnification (×4) hematoxylin-eosin image The image shows a granulomatous cellular infiltrate forming granulomas of the same age

**Figure 5:**
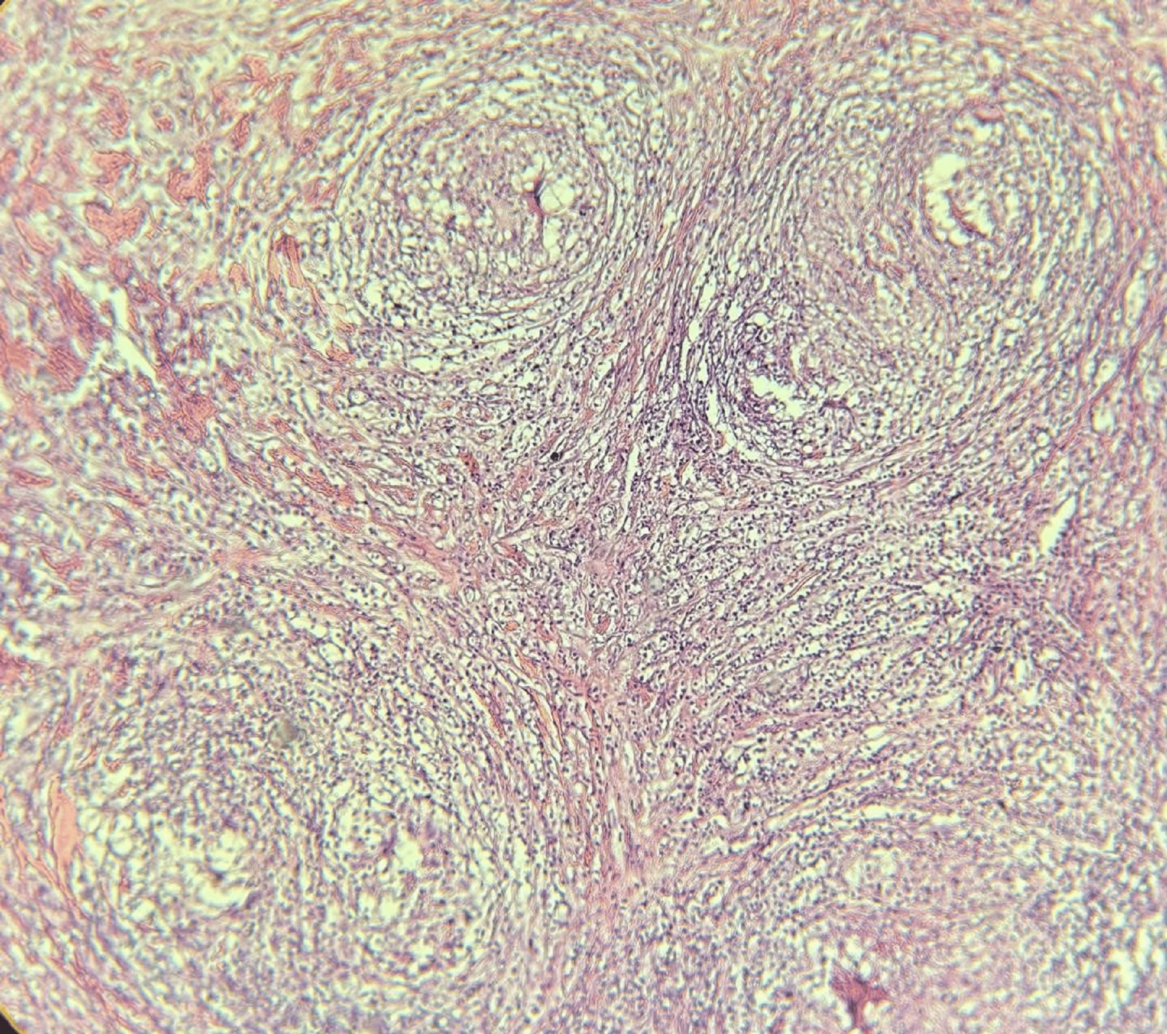
Hematoxylin-eosin image at magnification (×10) The image shows that the granulomas are made of epithelioid cells and Langhans-type giant cells on a fibrous background without necrosis and surrounded by lymphocytes

**Figure 6:**
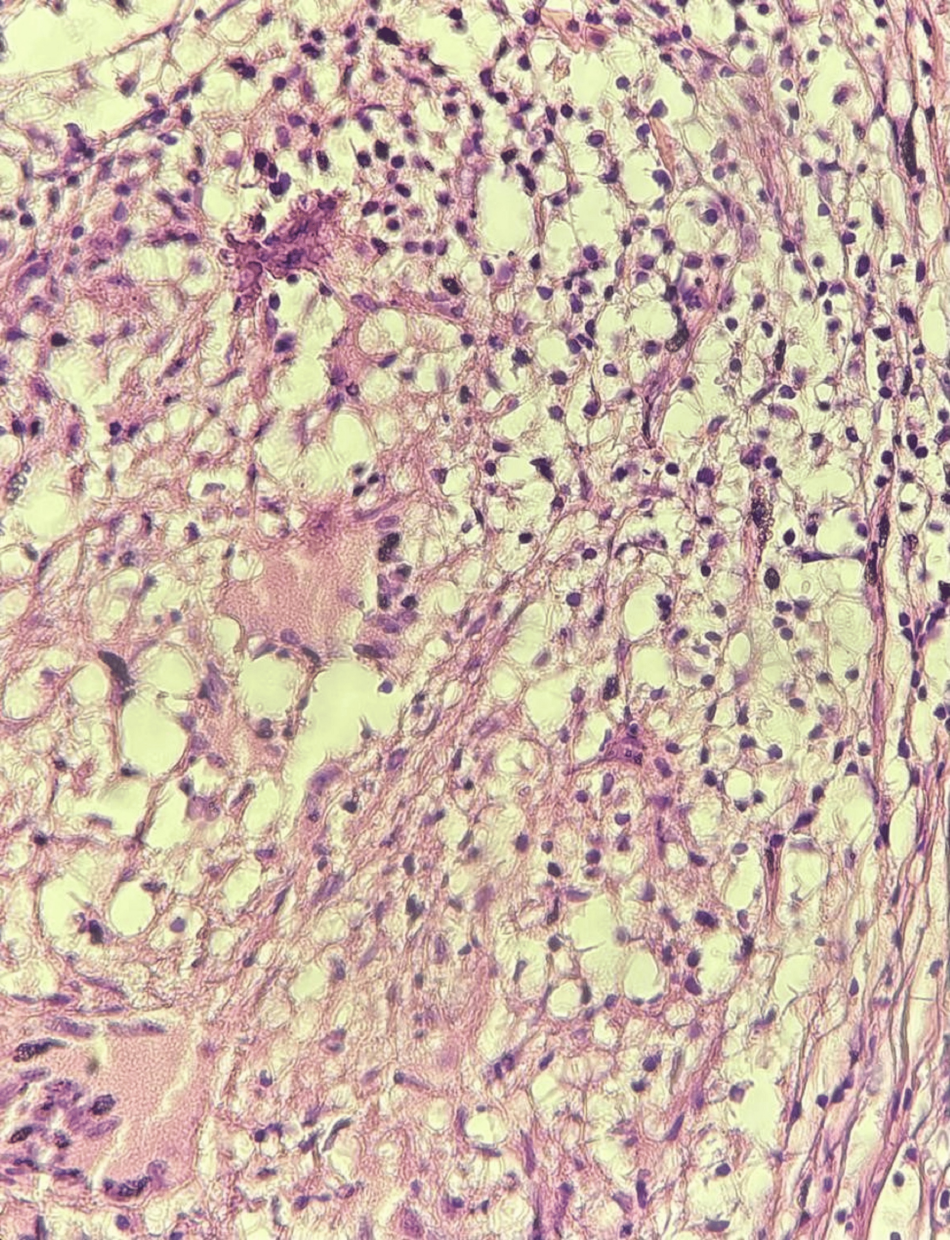
High-magnification (×40) hematoxylin-eosin image The image shows Langhans-type giant cells

A diagnosis of acute pericardial effusion was made. Given the size of the effusion and the need for a definitive tissue diagnosis, surgical pericardial drainage via a subxiphoid window was preferred over needle pericardiocentesis. Analysis of the pericardial fluid showed a hemorrhagic appearance and exudative with a protein level of 52 g/L (reference <30g/l) and hypercellularity with a predominance of lymphocytes at 93% (reference <50%). Pericardial fluid mycobacterial culture was negative, and PCR showed the absence of *Mycobacterium tuberculosis* DNA. GeneXpert test and adenosine deaminase for pericardial fluid were negative. Blood tests revealed a negative troponin level and a positive CRP level. Liver and kidney functions were normal (Table 1). The serology test for hepatitis viruses was negative. HIV serology was negative. Pericardial biopsy revealed a giant cell granuloma without caseous necrosis. Specific stains for mycobacterial and fungal infections were negative (Figures 4-6).

**Table 1:** Pericardial fluid analysis and blood tests

Pericardial fluid analysis and blood test results		
Pericardial fluid	Values	References
Appearance	Hemorrhagic	Clear
Proteins	52 g/l	< 30 g/L
Total nucleated cells	1126/µl	< 1000/µl
Leukocytes	1125/µl	< 500/µl
Lymphocytes	93.90%	< 50%
Neutrophils	6.10%	<4.5%
Red blood cells	24,748 10^3^/µl	Absence of red blood cells
Epithelial cells	1	< 40/µl
Cultures in common media	Negative	Negative
PCR	Absence of Mycobacterium tuberculosis DNA	
GeneXpert	Negative	Negative
Adenosine deaminase	10	0–40 U/L
Blood test		
Alanine aminotransferase	29	7–56 u/L
Aspartate aminotransferase	20	10–40 U/L
Alkaline phosphatase	64	44–147 U/L
Albumin	3	3.5–5.5 g/dL
Bilirubin (total)	0.6	0.1–1.2 mg/dL
Gamma-glutamyltransferase (GGT)	23	9–48 U/L
Total protein	7	6.0–8.3 g/dL
Prothrombin time	11	10.9–12.5 seconds
Estimated glomerular filtration rate	110	> 90 mL/min/1.73m²
Glycemia	4.2	3.9–5.5 mmol/L
Calcemia	9	8.5–10.5 mg/dL
Vitamin D	36	30 to 50 ng/mL
Hemoglobin	15	13.8 to 17.2 g/dL
Platelets	4,00,000	150,000 – 450,000/ µl
High-sensitivity troponin	3	< 22 ng/L
C-reactive protein	12	< 1 mg/dL
Sodium	140	135–145 mEq/L
Potassium	4	3.5–5.0 mEq/L
Chloride	98	96–106 mEq/L

The combination of negative molecular testing and the strict absence of caseous necrosis on histology allowed tuberculosis to be excluded. A diagnosis of cardiac sarcoidosis was made. The search for other manifestations of sarcoidosis was negative. The chest CT scan showed no pulmonary or mediastinal involvement. Biological tests were performed, which revealed normal calcium and vitamin D levels (Table 1). Serum angiotensin-converting enzyme testing was not available at our hospital.

The patient was started on corticosteroid therapy (prednisone 0.5 mg/kg/day) for six weeks, followed by a 12-month tapering phase. Acute pericarditis was treated with aspirin 1000 mg three times daily for three weeks, with a gradual taper over six weeks, combined with colchicine 0.5 mg twice daily for three months. Atrial fibrillation spontaneously returned to sinus rhythm. Anticoagulation with rivaroxaban was initiated because the CHA₂DS₂-VA score was 2. The one-year follow-up was favorable, with no recurrence of the pericardial effusion.

## Discussion

Sarcoidosis is a rare disease with an unknown cause. Several pathophysiological hypotheses have been proposed, including the presence of infectious or environmental triggers, genetic factors, and autoimmune responses [2]. Cardiac sarcoidosis primarily affects the myocardium [4]. Pericardial involvement in sarcoidosis most often manifests as a minimal effusion. It is not included in the diagnostic algorithms for sarcoidosis, likely due to underreporting of cases. However, pericarditis is a rare presentation of cardiac sarcoidosis [10]. A recent literature review identified only 31 cases of pericardial sarcoidosis reported since 1966, two of which did not involve extracardiac sarcoidosis [5].

In our patient, the diagnosis of sarcoidosis was confirmed by the presence of a granuloma lacking caseous necrosis on pericardial biopsy, with the exclusion of infectious causes based on a comprehensive etiological evaluation of the pericardiocentesis fluid, including mycobacterial culture, GeneXpert, and PCR. The search for extracardiac involvement did not reveal any pulmonary or mediastinal sarcoidosis. The presence of sterile, exudative, and hemorrhagic pericardial fluid in cases of sarcoidosis has been described in some clinical cases [11].

Pericardial biopsy is a safe procedure, but its role in the etiological evaluation of pericarditis remains controversial [12]. In our case, the pericardial biopsy revealed a noncaseating granuloma. Current statements recommend cardiac MRI and fluorodeoxyglucose PET as the imaging modalities of choice for detecting and quantifying myocardial involvement in suspected cardiac sarcoidosis [3]. Unfortunately, these advanced imaging techniques were unavailable at our center in Nouakchott, which limited our evaluation to transthoracic echocardiography, which has a lower sensitivity for early myocardial disease.

The therapeutic management of cardiac sarcoidosis relies on immunosuppressive therapy and the stratification of arrhythmic risk, which guides decisions regarding implantable cardiac device therapy. Corticosteroid therapy remains the first-line treatment for cardiac sarcoidosis [3]. Our patient was started on long-term corticosteroid therapy, and the short-term outcome was favorable. This report describes an unusual clinical presentation of cardiac sarcoidosis and highlights the value of pericardial biopsy in the evaluation of acute pericarditis.

## Conclusions

Acute pericardial sarcoidosis is a rare manifestation of cardiac sarcoidosis. Guidelines do not include pericardial sarcoidosis in diagnostic algorithms, even though pericardial involvement may be the initial manifestation of systemic sarcoidosis. Diagnosing pericardial sarcoidosis is difficult and is most often based on evidence of extracardiac involvement. This clinical case report highlights the value of pericardial biopsy in the etiological evaluation of acute pericarditis with effusion and emphasizes the need to consider sarcoidosis in cases of unexplained pericardial disease.
